# Autophagy is required for crizotinib-induced apoptosis in MET-amplified gastric cancer cells

**DOI:** 10.18632/oncotarget.18386

**Published:** 2017-06-07

**Authors:** Rebecca D. Schroeder, Woonyoung Choi, David S. Hong, David J. McConkey

**Affiliations:** ^1^ Department of Investigational Cancer Therapeutics, The University of Texas MD Anderson Cancer Center, Houston, Texas, USA; ^2^ Department of Urology, The University of Texas MD Anderson Cancer Center, Houston, Texas, USA; ^3^ The University of Texas MD Anderson Cancer Center UTHealth Graduate School of Biomedical Sciences, Houston, Texas, USA; ^4^ Experimental Therapeutics Academic Program, Houston, Texas, USA

**Keywords:** MET amplification, autophagy, resistance mechanism, gastric cancer

## Abstract

MET amplification has been clinically credentialed as a therapeutic target in gastric cancer, but the molecular mechanisms underlying sensitivity and resistance to MET inhibitors are still not well understood. Using whole-genome mRNA expression profiling, we identified autophagy as a top molecular pathway that was activated by the MET inhibitor crizotinib in drug-sensitive human gastric cancer cells, and functional studies confirmed that crizotinib increased autophagy levels in the drug-sensitive cells in a concentration-dependent manner. We then used chemical and molecular approaches to inhibit autophagy in order to define its role in cell death. The clinically available inhibitor of autophagy, chloroquine, or RNAi-mediated knockdown of two obligate components of the autophagy pathway (ATG5 and ATG7) blocked cell death induced by crizotinib or RNAi-mediated knockdown of MET, and mechanistic studies localized the effects of autophagy to cytochrome c release from the mitochondria. Overall, the data reveal a novel relationship between autophagy and apoptosis in gastric cancer cells exposed to MET inhibitors. The observations suggest that autophagy inhibitors should not be used to enhance the effects of MET inhibitors in gastric cancer patients.

## INTRODUCTION

Although our understanding of significant risk factors and screening methods for gastric cancer has improved over the last decade, it remains the fourth most prevalent cancer and second leading cause of cancer-related death worldwide [1, 2]. The frontline treatment strategies for gastric cancer are surgical resection and chemotherapy, but because most patients have advanced-stage tumors at diagnosis, the 5-year survival rate is less than 30% [3–5]. Recent genomics studies identified several genes related to receptor tyrosine kinase (RTK) signaling as candidate therapeutic targets in gastric cancer [6, 7]. Among them, MET is of particular interest because recent clinical trials demonstrated that MET inhibitors had significant clinical activity [8, 9], particularly in patients whose tumors contained MET amplification [10–12], and preclinical studies indicated that MET amplification usually created MET dependency in gastric cancer cells [13, 14]. Multiple studies annotated the frequency of MET amplification in gastric cancers and reported rates of 9-30% [8, 9, 15], with high-level MET amplification in about 4% (12/287) of tumors [7].

Although MET inhibitors have clinical activity, not all MET-amplified tumors respond, and a deeper understanding of the molecular determinants of response and resistance is therefore crucial. MET inhibitors have both cytotoxic and cytostatic effects in MET-amplified cells, and mechanistic studies implicated upregulation of the pro-apoptotic BCL2 family protein BIM and downregulation of several pro-survival genes, including the IAP family members c-IAP1, XIAP, and survivin, in cell death [14]. Other studies demonstrated that MET inhibitors caused increases in autophagy and autophagy-associated gene expression, but whether autophagy promoted cell death or cell survival was not determined [16, 17]. Autophagy inhibitors promoted cell death in cancer cells exposed to other pro-apoptotic agents [18, 19], raising the possibility that autophagy inhibitors might promote the effects of MET antagonists in human gastric cancer cells. We therefore designed the present study to determine whether we could reproduce the previous observations [16, 17] and to evaluate the effects of blocking autophagy on MET inhibitor-induced cell death.

## RESULTS

### Effects of the MET inhibitor crizotinib on human gastric cancer cells

**Figure 1:**
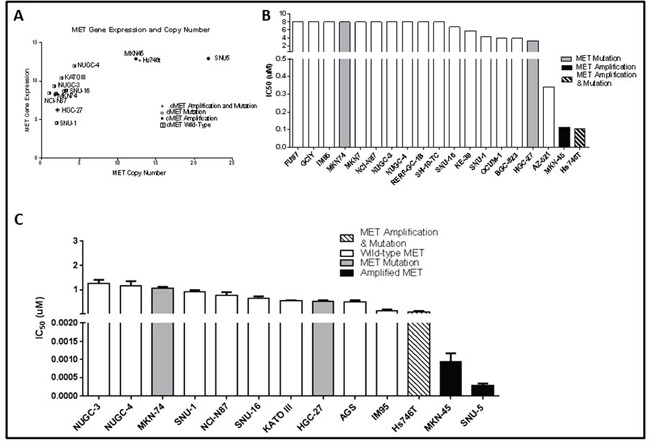
MET amplified gastric cancer cells undergo growth arrest when exposed to MET inhibitor crizotinib **(A)** MET RNA expression levels were compared to MET copy numbers for a panel of thirteen gastric cancer cell lines using data generated by the CCLE. **(B)** Inhibitory concentration 50% (IC50) values of crizotinib in a panel of nineteen gastric cancer cell lines were compared using data generated by the CCLE. **(C)** Independently determined IC50 values of crizotinib in a panel of thirteen human gastric cancer cell lines. Cells were exposed to increasing concentrations of crizotinib for 5 days and viable cells were assessed using the MTT colorimetric assay. Data are means ± SEM from three independent experiments.

**Figure 2:**
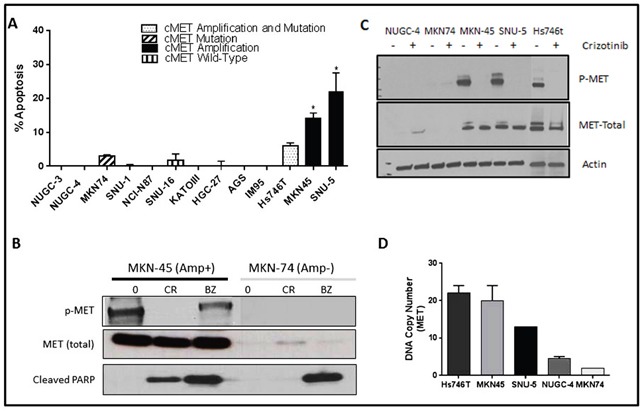
MET amplified gastric cells undergo apoptosis when exposed to crizotinib **(A)** Thirteen human gastric cancer lines were incubated with or without 10nM crizotinib for 48 hours, and DNA fragmentation characteristic of apoptosis was measured by propidium iodide (PI) staining and FACS analyses. Values represent normalized results subtracting untreated controls, which were all less than 15%. Student t test, *p≤0.005 crizotinib versus untreated control. **(B)** One MET amplified and one non-amplified cell line were incubated with or without 100nM crizotinib or 1uM bortezomib for 48 hours. The cells were then lysed and total protein was extracted using RIPA lysis buffer. Total lysates were then subjected to immunoblotting analysis using anti-phospho-MET, anti-total-MET, and anti-cleaved-PARP antibodies. **(C)** Three MET amplified (Hs746t, SNU-5 and MKN45) and two non-amplified cell lines (MKN74 and NUGC-4) were incubated with or without 100nM crizotinib 24 hours, and total protein was extracted using RIPA lysis buffer. Total lysates were then analyzed by immunoblotting using anti-phospho-MET, anti-total-MET, and β-actin antibodies. **(D)** MET DNA copy numbers were analyzed using TaqMan copy number assays. Three cell lines Hs746t, SNU-5 and MKN45 had high level MET amplification with average copy numbers of 22, 13 and 20, respectively. Data are means ± SEM from two biological replicates.

As a first step in defining the determinants of MET dependence, we correlated the IC_50_ values for the MET inhibitor crizotinib with MET gene expression and amplification using publicly available data from the Cancer Cell Line Encyclopedia (CCLE) project (Figure 1) [20]. Overall, there was a good correlation between MET mRNA expression and MET copy numbers in the cell lines (Figure 1A). Of the 19 available gastric cell lines, only two (MKN45 and Hs746t) contained amplified MET, and they were also the only cell lines that the CCLE team found to be sensitive to crizotinib at IC_50_ levels less than the peak plasma concentration obtained in patients, 57 nM (Figure 1B) [21]. We then attempted to confirm the results in an independent (but overlapping) panel of 13 gastric cancer cell lines. The only cell lines that were sensitive to clinically achievable concentrations of crizotinib were two of the three that contained amplified MET (SNU-5 and MKN45), whereas the Hs746t cells were more resistant (Figure 1C). Next, we examined the effects of crizotinib on apoptosis in the same panel of 13 gastric cancer cell lines (Figure 2). Consistent with the MTT results, only the SNU-5 and MKN45 cell lines displayed significant crizotinib-induced increases in apoptosis-associated DNA fragmentation, as measured by propidium iodide staining and FACS analysis (Figure 2A) [13, 14, 22], and we confirmed these effects using an independent apoptosis assay (immunoblotting for cleaved poly-(ADP-ribose) polymerase (PARP)) (Figure 2B). Consistent with the CCLE's mRNA expression results (Figure 1A), phosphorylated and total MET levels were higher in the Hs746t, SNU-5, and MKN45 cells that contained amplified MET as compared to the levels observed in the NUGC-4 or MKN74 cells that contained wild-type or mutated MET, respectively (Figure 2C). We also independently confirmed the CCLE's observation that MET was highly amplified (≥10 copies) in the Hs746t, MKN45, and SNU-5 cells (Figure 2D).

### Effects of crizotinib on global gene expression

**Figure 3:**
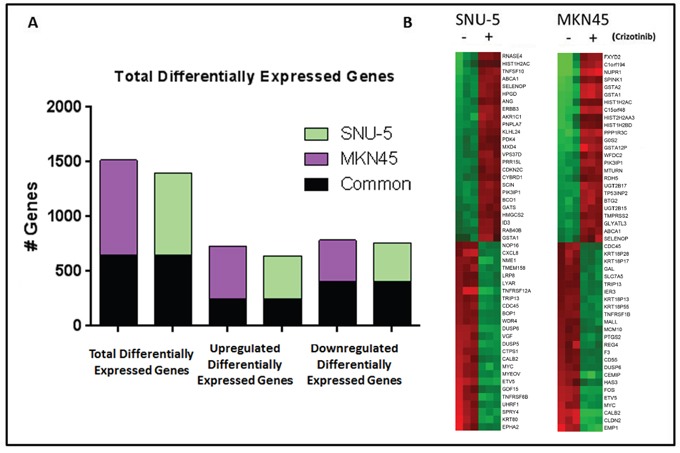
MET inhibition results in significant changes in gene expression in MET amplified gastric cancer cell lines **(A)** Graph representing the total number of genes that had a P<0.001 with FDR <0.05 and 2-fold cut-off in the two MET amplified cell lines following incubation with crizotinib for 24 hours (left). The total number of genes up-regulated (middle) and down-regulated (right) following incubation with crizotinib for 24 hours, with the number of common genes between the two cell lines shaded in black. **(B)** Heat map of the top 50 differential expressed genes for each cell line following incubation with crizotinib for 24 hours. Red, higher relative expression; green, lower relative expression.

To define the molecular mechanisms involved in cell death, we incubated the inhibitor- sensitive lines with or without crizotinib for 24 h and performed whole-genome mRNA expression profiling (Figure 3). In both cell lines, >1400 genes were differentially expressed under the two conditions (Figure 3A). We observed substantial overlap between the two cell lines with regard to the genes that were differentially expressed following incubation with crizotinib – 406 downregulated and 246 upregulated genes were shared (Figure 3A). We extracted the top 25 upregulated and downregulated transcripts in each cell line and observed that a number of genes related to cell death and cell growth/proliferation were enriched in them (Figure 3B). In control experiments, we confirmed that top differentially expressed genes related to cell death and cell growth/proliferation identified by gene expression profiling were also differentially expressed when they were measured by RT-PCR (Supplementary Figure 1A, 1B) or immunoblotting (Supplementary Figure 1C) in the same cell lines.

**Figure 4:**
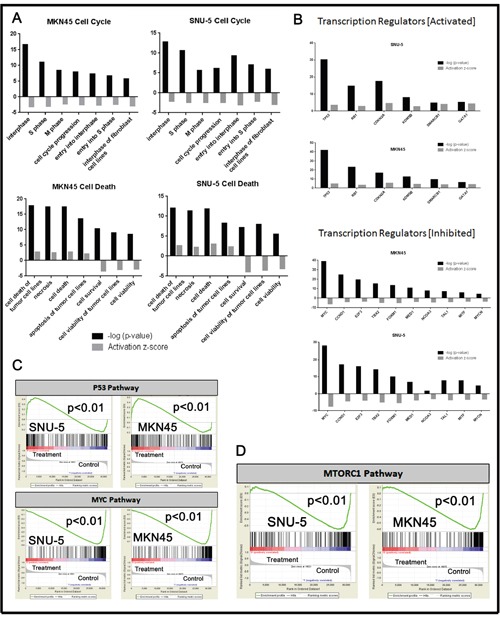
Pathway analyses of crizotinib-induced changes in gene expression **(A)** Significant changes in gene expression were analyzed using Ingenuity Pathway Analysis (IPA) using the molecular and cellular functions of the platform. **(B)** Significantly activated/inhibited transcriptional regulators from whole genome mRNA expression profiling using IPA. (**C** and **D**) Selection of the top common results within GSEA H: hallmark gene sets from the molecular gene sets database are displayed for each of the cell lines. All results within the GSEA analysis have a P<.01 and FDR <.05.

**Figure 5:**
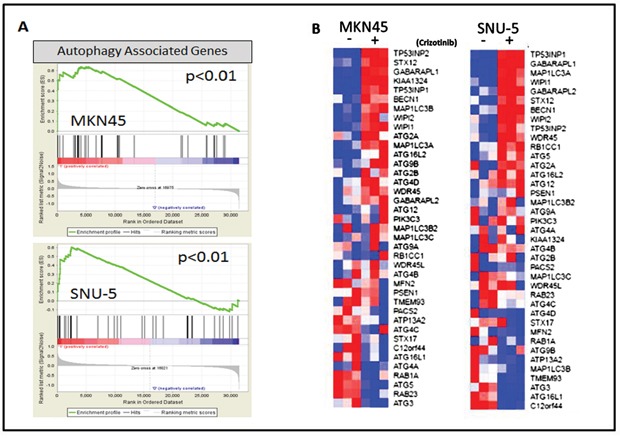
MET inhibition stimulates an autophagy gene expression signature **(A)** Gene set enrichment analysis (GSEA) was used to determine whether a gene expression signature associated with autophagy (BP: GO biological process gene set) was stimulated by crizotinib in the SNU-5 (P<.01, FDR= .04) and MKN45 (P<.01, FDR= .07) cells. **(B)** Expression of the top differentially expressed autophagy-associated genes included in the GSEA data set (GO autophagosome organization) used in panel A. The heat maps depict relative expression of autophagy markers in two MET amplified cell lines following incubation with crizotinib for 24 hours. Red, higher relative expression; blue, lower relative expression.

To more clearly define the pathways that were altered by drug exposure we used the “molecular and cellular functions” function in Ingenuity Pathway Analysis (IPA, Ingenuity Systems; http://www.ingenuity.com) to analyze the gene expression profiling data (Figure 4). Molecular changes related to cell death, cell cycle (Figure 4A) and cell growth and proliferation (Supplementary Figure 2A) were among the top alterations observed in both MET-amplified cell lines. We extracted the top inhibited and activated transcriptional regulators for each cell line and observed extensive overlap between the two cell lines (Figure 4B). The activated population was highly enriched with transcription factors involved in cell cycle/proliferation arrest and apoptosis activation, whereas the inhibited population was enriched for transcription factors involved in cell cycle progression and cellular proliferation. Additionally, cellular growth and proliferation, cell cycle, cell death and survival were among the top five molecular and cellular functions modulated in each cell line (Supplementary Figure 2B). We confirmed these results with gene set enrichment analyses (GSEA) by using previously identified gene sets available at molecular signature database (MSigDB; www.broadinstitute.org/gsea/). Initially, we queried our data using the H: hallmark resource containing 50 gene sets. P53 signaling was positively enriched and MYC signaling was negatively enriched after crizotinib exposure in both cell lines (Figure 4C), and MTORC1 signaling was downregulated by crizotinib (Figure 4D). MTORC1's ability to suppress autophagy is well established, and inhibition of MTORC1 induces autophagy [23, 24]. This finding prompted us to query the BP: GO resource containing 4653 gene sets identified by the Gene Ontology Consortium (GO) as enriched in various biologic processes (Figure 5). These analyses identified autophagy as among the top thirty biologic processes enriched among the differentially expressed genes in both of the two MET-amplified cell lines (Figure 5A). We also observed concordance in the modulation of the top autophagy-related gene set in both cell lines (Figure 5B).

### Effects of autophagy inhibition on apoptosis

**Figure 6:**
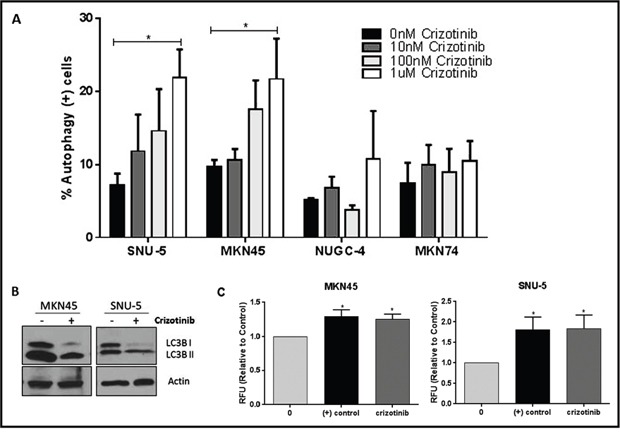
MET inhibition induces autophagy in the crizotinib-sensitive cells **(A)** Crizotinib induces autophagy in a concentration-dependent fashion. The indicated human gastric cancer cell lines were incubated with increasing concentrations of crizotinib (0, 10, 100, 1000nM) for 72 hours and autophagy was accessed by acridine orange staining coupled with flow cytometry. The SNU-5 and MKN45 cells contain high level MET amplification, whereas the NUGC-4 and MKN74 cells do not. **(B)** Crizotinib induces LC3-II processing. SNU-5 and MKN45 cells were incubated with 100nM crizotinib with for 24hrs and LC3 expression was analyzed by Western blotting. **(C)** Crizotinib causes increased autophagic flux in the drug-sensitve cells. SNU-5 and MKN45 cells were incubated with 100nM crizotinib with for 24hrs and autophagy was accesses by fluorescence microplate reader with the application of Cyto-ID autophagy detection kit. Data are means ± SEM from three independent experiments. Student *t* test, *p≤0.05.

We used functional assays to attempt to confirm the gene expression profiling results using two of the drug-resistant lines (NUGC-4, MKN74) as controls (Figure 6). We exposed the cells to increasing concentrations of crizotinib for 72 hours and then measured autophagy by acridine orange staining and FACS analysis [25–27]. Crizotinib caused concentration-dependent increases in autophagy in both MET-amplified cell lines but not in the drug-resistant cells (Figure 6A). We confirmed these results by immunoblotting to detect conversion of LC3B I to LC3II (Figure 6B), and by measuring autophagic flux using an cationic amphiphilic tracer (CAT) dye that rapidly partitions into cells in a similar manner as drugs that induce phospholipidosis (from Enzo Life Sciences, Inc) (Figure 6C).

**Figure 7:**
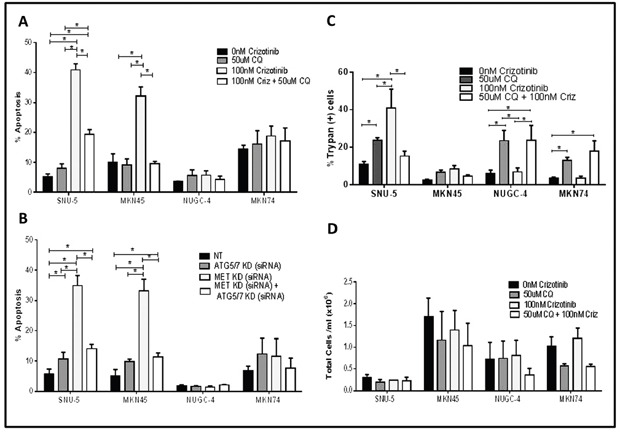
Autophagy is required for crizotinib-induced apoptosis (**A** and **B**) Effects on apoptosis. **(A)** Gastric cancer cell lines (SNU-, MKN45, MKN74, and NUGC-4) were incubated with or without 100nM crizotinib, 50uM chloroquine (CQ) and 100nM crizotinib + 50uM chloroquine for 48 hours and PI-FACS was used to quantify cells with fragmented DNA due to apoptosis. Student t test, *p≤0.0005. **(B)** Gastric cancer cell lines (SNU-5, MKN45, MKN74, and NUGC-4) were transfected with a non-targeting (NT) or MET siRNA, ATG5/7siRNA or MET siRNA + ATG5/7 siRNA for 48 hours and PI-FACS was used to quantify apoptotic cell death. (**C** and **D**) Effects on necrosis. Gastric cancer cell lines (SNU-, MKN45, MKN74, and NUGC-4) were incubated with or without 100nM crizotinib, 50uM chloroquine (CQ) or 100nM crizotinib + 50uM chloroquine for 72 hours and trypan blue exclusion/ ViCELL was used to quantify total cell death and absolute cell numbers. Data are means ± SEM from three independent experiments. Student *t* test, *p≤0.05.

Depending on the specific biological context, autophagy can either promote or inhibit cancer cell death [18, 28]. Therefore, we designed mechanistic experiments to define the role of autophagy in crizotinib-induced cell death (Figure 7). First, we examined the effects of blocking autophagy with chloroquine, a clinically approved anti-malarial drug that inhibits autophagy by raising lysosomal pH [29, 30]. Chloroquine did not induce statistically significant increases in the levels of apoptosis in any of the cell lines on its own (Figure 7A). However, chloroquine did induce significant increases in the numbers of trypan blue-positive (necrotic) cells in all of the cell lines except for the MET-amplified cell line MKN45 (Figure 7C). On the other hand, chloroquine caused a statistically significant decrease in crizotinib-induced apoptosis in the MET amplified cell lines (MKN45 and SNU-5) (Figure 7A). To confirm that these effects were caused by autophagy inhibition, we used RNA interference to knock down two obligate components of the autophagy pathway (ATG5 and ATG7) and/or MET, and we examined the effects of molecular interruption of autophagy on apoptosis induced by MET knockdown. ATG5/7 knockdown blocked MET knockdown-induced apoptosis in both of the crizotinib-sensitive cell lines (Figure 7B). We also measured total cell numbers following exposure to chloroquine with or without crizotinib and did not observe statistically significant decreases in cell numbers in any of the cell lines (Figure 7D). Therefore, the decreased apoptosis in the chloroquine-exposed MKN45 and SNU-5 cells was not caused by a decrease in total cell numbers.

**Figure 8:**
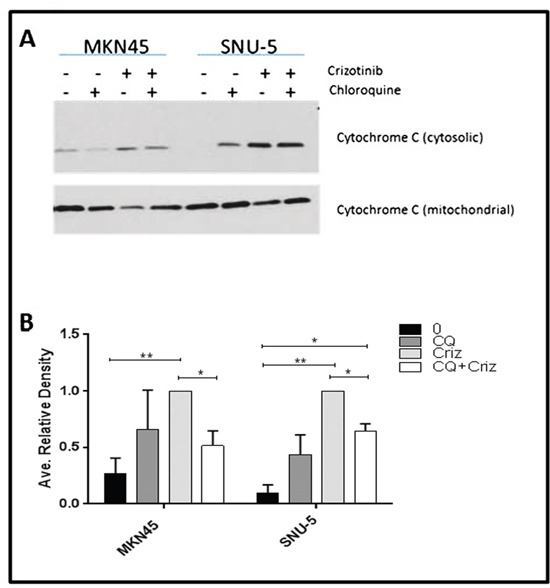
Autophagy is required for crizotinib-induced cytochrome c release **(A)** Measurement of cytochrome c release by immunoblotting. The MET-amplified, crizotinib-sensitive gastric cancer cell lines (SNU-, MKN45) were incubated with or without 100nM crizotinib, 50uM chloroquine (CQ) and 100nM crizotinib + 50uM chloroquine for 6 hours and western blot analysis was used to quantify cytochrome c levels in the cytosol and mitochondria. **(B)** Quantitative densitometry of the protein expression of the cytosolic fraction versus the total cytochrome c content (cytosolic plus mitochondrial fractions) of cytochrome c in each sample. Data are means ± SEM from three independent biological replicates. Student t test, *p≤0.05 **p≤0.005.

Cytochrome c release from mitochondria is a central commitment point for apoptotic cell death. We therefore wondered whether autophagy inhibition might attenuate apoptosis by preventing crizotinib-induced cytochrome c release (Figure 8). To test this hypothesis, we incubated the SNU-5 or MKN45 cells with crizotinib with or without chloroquine for 6 h and measured cytosolic cytochrome c levels by immunoblotting as described previously [31]. Chloroquine caused statistically significant inhibition of cytochrome c release in both cell lines (representative immunoblots are displayed in Figure 8A, and the results of 3 independent experiments are quantified in Figure 8B).

### Autophagy inhibition does not confer resistance to other therapeutic agents

Finally, we wondered whether the requirement for autophagy for apoptosis was specific for crizotinib or might also be observed with other stimuli. To address this question, we examined the effects of chloroquine on apoptosis induced by two alternative therapeutic agents (cisplatin and bortezomib) (Supplementary Figure 3). Clinically achievable concentrations of bortezomib induced significant increases in apoptosis in three of the four cell lines (MKN45, MKN74, and NUGC-4) (Supplementary Figure 3A). Chloroquine caused a modest but statistically significant decrease in bortezomib-induced apoptosis only in the MKN45 cells at the highest concentration of bortezomib (100nM, p=0.04) (Supplementary Figure 3A). Cisplatin also increased apoptosis in all four of the cell lines (Supplementary Figure 3B). Again, chloroquine caused modest (and not statistically significant) decreases in cisplatin-induced apoptosis only in the MKN45 cells (Supplementary Figure 3B). Together, the results support the conclusion that the requirement for autophagy is relatively selective for crizotinib-induced apoptosis.

## DISCUSSION

MET amplification is a clinically validated therapeutic target in gastric cancer [32, 33]. Here we demonstrate that MET-amplified gastric cancer cells exhibited growth arrest and cell death in response to incubation with the MET inhibitor crizotinib, whereas MET inhibition had no significant effects in cells without MET amplification, irrespective of whether they had high MET mRNA expression or contained MET mutations. Our results are consistent with previous preclinical observations [13, 14] and recent clinical experience [10, 33–35]. Nevertheless, it was surprising to us that MET inhibitors had no measurable effects in the gastric cancer cell lines that contained activating MET mutations. Perhaps MET mutations control some aspect of cancer biology that was not measured in this and the other preclinical studies that have been performed to date. Alternatively, it is possible that activating MET mutations act at an early stage in tumor progression and/or become less important in established human gastric cancer cell lines.

A top priority for ongoing investigation is to identify strategies that increase MET inhibitor sensitivity and overcome the development of the acquired resistance that is likely to emerge following prolonged MET inhibition. Using whole-genome mRNA expression profiling, we observed that genes involved in the regulation of autophagy were modulated in both MET inhibitor-sensitive cell lines, and GSEA confirmed that genes associated with autophagy were highly enriched following MET inhibition. Furthermore, direct measurements of autophagy confirmed that crizotinib induced concentration-dependent increases in both cell lines, consistent with previous reports [16, 17]. Autophagy is a complex process that mediates a variety of different physiological functions, including degrading dysfunctional cellular components, protection of organelle function, promoting cell survival, decreasing metabolic stress, and executing apoptosis [18, 36]. With regard to cell death, the effects of autophagy are context-dependent, resulting in cytoprotective or cytotoxic effects depending on the specific physiological or pathological context [18, 19, 37]. Because of this complexity, and the interest in targeting autophagy to improve the effects of cancer therapies, it was important to understand what role autophagy activation played in apoptosis induced by MET inhibition in the MET-amplified gastric cancer cells [17, 37, 38]. In both of the drug-sensitive cell lines (MKN45 and SNU-5), autophagy inhibition caused statistically significant decreases in MET inhibitor-induced apoptosis, regardless of the approach employed to inhibit autophagy (i.e. direct siRNA-mediated knockdown or chemical inhibition). Our preliminary analyses of the molecular mechanisms involved demonstrated that autophagy was required for crizotinib-induced cytochrome c release, considered the proximal commitment point for apoptosis in most examples of the response.

Although these results were surprising to us, they are not unprecedented. Past studies also demonstrated that autophagy was essential for apoptosis [18, 39, 40], and another group concluded that autophagy was required for cytochrome c release [41], and additional mechanistic studies are required to determine precisely how autophagy promotes cytochrome c release in gastric cancer cells exposed to MET inhibitors. Overall, our results demonstrate that autophagy inhibitors do not potentiate MET inhibitor-induced apoptosis in gastric cancer cells. Clinically, our findings underscore the importance of understanding tumor biology prior to launching trials of combination therapies with autophagy modulators and growth factor receptor inhibitors in patients.

## MATERIALS AND METHODS

### Cell lines and culture

MKN45, MKN74, NUGC-3, NUGC-4, and IM95 gastric cancer cells were a gift from Julie G. Izzo, M.D., Department of Experimental Therapeutics, MD Anderson. KATOIII, NCI-N87, SNU-16, SNU-5, AGS, Hs746t, and SNU-1 gastric cancer cells were obtained from American Type Culture Collection (Manassas, VA). HGC-27 gastric cancer cells were obtained from Sigma-Aldrich (St. Louis, MO). All cells were validated by DNA fingerprinting using AmpFlSTR^®^ Identifiler^®^ Amplification kit (Applied Biosystems, Foster City, CA), performed by the MD Anderson Characterized Cell Line Core. All gastric cancer cells (except for SNU-5) were maintained in RPMI-1640 medium supplemented with 10% FBS (HyClone/Thermo Scientific, Waltham, MA), minimum essential medium (MEM) vitamins, sodium pyruvate (Mediatech/Corning Cellgro, Manassas, VA), L-glutamine, non-essential amino acids, penicillin/streptomycin (Lonza, Switzerland), and HEPES buffer. SNU-5 cells were cultured in Iscove's modified Dulbecco's medium supplemented with 10% FBS, MEM vitamins, L-glutamine, sodium pyruvate, non-essential amino acids, penicillin/streptomycin, and HEPES. All cells were grown at 37° C in 5% CO_2_.

### Chemicals and antibodies

Crizotinib was purchased from Selleck Chemicals (Houston, TX). Bortezomib was purchased from ChemieTek (Indianapolis, IN). Cisplatin was purchased from EMD Millipore Corp (Billerica, MA). Propidium iodide, oligomycin A, chloroquine and acridine orange were purchased from Sigma-Aldrich (St. Louis, MO). The following antibodies were purchased from the indicated sources: MET, p-MET, MYC, cleaved PARP (Cell Signaling Technology, Beverly, MA); Cytochrome C and LC3 (BD Pharmingen); and β-actin (Sigma-Aldrich).

### Cell viability assay

Cell death was measured using the Vi-CELL XR Cell Viability Analyzer (Beckman Coulter) following incubation with the inhibitor for 48 hours. This analyzer processes and analyzes cells using the trypan blue dye exclusion method.

### Cell proliferation assay

Growth arrest was quantified using a 5- day dimethyl thiazolyl diphenyl tetrazolium salt (MTT) assay as previously described [42].

### Immunoblotting analyses

Cells were harvested by scrapping and lysed in buffer containing 50mM Tris-HCL, 150mM NaCl, 1mM EDTA, 1% Triton X-100, 1% sodium deoxycholate, 0.01% SDS, 2mM sodium orthovanadate (Na3VO4), 1mM NaF, 1mM Glycophosphate, 1mM PMSF and Complete Mini protease/ phosphatase inhibitor tablets (Sigma-Aldrich). Protein concentrations were measured using the BCA Protein Assay Kit (Thermo Scientific, Rockford, IL). Lysates were boiled in sample buffer (62.5 mmol/L Tris-HCl (pH 6.8), 10% (w/v) glycerol, 100 mmol/L DTT, 2.3% SDS, 0.002% bromophenol blue) for 5 minutes and cooled at room temperature for 10 minutes. Samples were then separated on 2-12% gradient SDS-PAGE gels at 100 V in electrophoresis buffer (25 mmol/L Tris-HCl (pH 8.3), 192 mmol/L glycine, 0.1% SDS) and then electrophoretically transferred onto nitrocellulose membranes in transfer buffer (25 mmol/L Tris-HCl, 192 mmol/L glycine, 20% methanol) overnight at 10 mV. The membranes were incubated in blocking buffer (5% nonfat milk in PBS) for 1 hour at room temperature while shaking. The membranes were then rinsed with PBS containing 0.1% Tween-20. The membranes were incubated with primary antibodies diluted 1:1000 in 1% milk overnight, washed, and then incubated with second antibodies (anti-mouse or anti-rabbit immunoglobulin) diluted 1:10,000 in 5% milk for 1 hour at room temperature while shaking. Immunoreactive proteins were detected using enhanced chemiluminescence (Amersham Biosciences, Piscataway, NJ) [43].

### Cytochrome c release assay

Release of cytochrome c from the mitochondria was measured by immunoblotting as previously described [31]. Cells were incubated with or without 100 nM crizotinib, 50uM chloroquine, or 100 nM crizotinib + 50 uM chloroquine for 6 hours. The cells were then obtained by scraping followed by gentle centrifugation at 1700rpm for 3 minutes. The pellets were then washed with cold PBS and re-spun for 3 minutes. Next the cells were lysed in an ice-cold buffer containing 250 mM Sucrose, 1 mM EDTA, 25 mM Tris, pH 6.8, 0.05% IGEPAL and a Complete Mini protease inhibitor tablet (Sigma-Aldrich) until the cells outer membrane was compromised as determined by the trypan blue exclusion assay. The cells were then centrifuged at 14,000 rpm for 5 min at 4 °C and the supernatant, containing the cytosolic fraction, was transferred to new tubes. The pellet containing the mitochondrial fraction was then suspended in lysis buffer, and cytochrome c was measured in each fraction by immunoblotting.

### Apoptosis detection

Apoptosis was quantified by propidium iodide staining coupled with flow cytometry as previously described [22]. The method involves propidium iodide staining of permeabilized cells; apoptotic cells release the DNA fragments produced as a consequence of the endogenous endonuclease activation that is associated with apoptosis, and they appear as hypodiploid cells when they are measured by flow cytometry.

### Copy number assay

DNA was isolated from gastric cell lines (MKN45, MKN74, NUGC-4, SNU-5 and Hs746t) using a genomic DNA extraction kit (Qiagen). *MET* gene copy number was determined using commercially available and pre-designed TaqMan Copy Number Assays (Applied Biosystems, Foster City, CA) as described previously [44]. The primer used for the *MET* gene was Hs05005660_cn. (Location: Chr.7:116778578 on GRCh38, Cytoband: 7q31.2). The *TERT* locus was used for the internal reference copy number. Real-time genomic PCR was performed in a total volume of 20μL in each well, which contained 10μL of TaqMan genotyping master mix and 20ng of genomic DNA and each primer. The PCR conditions were 95°C for 10 minutes, 40 cycles of 95°C for 15 seconds, and 60°C for 1 minute. Data were analyzed using SDS2.2 software and CopyCaller software (Applied Biosystems).

### Autophagy detection

Autophagy levels were measured using two distinct assays. The first method involved using a lysotropic dye, acridine orange, which accumulates in acidic organelles in a pH-dependent manner, becomes protonated and trapped, and emits a bright red fluorescence. The red fluorescence was then detected by fluorescence-activated cell sorting (Coulter, FL2 channel). Bafilomycin A1 (Sigma Chemical Co.) was dissolved in DMSO and added to the cells 30 min before the addition of acridine orange. The second method employed the use of the CYTO-ID^®^ Autophagy detection kit (Enzo Life Sciences, Inc) according to the manufacturer's protocol. Cells were then analyzed by fluorescence microplate reader.

### Gene knockdown

The siRNAs used were obtained through Dharmacon/Thermo Scientific; ON-TARGETplus SMARTpool siRNA reagent targeting *MET* proto-oncogene, receptor tyrosine kinase (*MET*) (catalog number L-003156-00) and non-targeting siRNA (catalog number D-001810-10-20). Lipofectamine RNAiMAX (Invitrogen/Life Techonologies, catalog no. 13778-075) was used to transfect the siRNAs into the cells.

### Gene expression profiling

Total RNA from cell pellets was isolated using the mirVana miRNA isolation kit (Ambion, Inc). RNA purity and integrity were measured by a NanoDrop ND-1000 spectrophotometer and Agilent Bioanalyzer, respectively, and only high-quality RNA was used for the cRNA amplification. The MET-amplified gastric cancer cell lines were analyzed by direct hybridization on Illumina Human HT12v4 chips (Illumina, San Diego, CA). Quantile normalization in the Linear Models for Microarray Data (limma) package in the R language environment was used to normalize the data. BRB Array Tools version 4.5.1 (National Cancer Institute) was used to analyze the data. The significantly differentially expressed genes (P<0.001 with FDR <0.05, 2-fold cut-off) were then extracted using class comparison tools with random variance t test to yield 1734 differentially expressed probes for SNU-5 representing 1405 genes and 1919 differentially expressed probes for MKN45 representing 1517. Gene expression profiling data was uploaded to Gene Expression Omnibus with accession number GSE77320.

### Pathway analyses

Functional and pathway analyses were performed using Ingenuity Pathway Analysis (IPA) software (Ingenuity^®^ Systems, CA), which contains a database for identifying networks and pathways of interest in genomic data. Based on the IPA knowledge database, p values and Z-scores can be calculated based on how many targets of each transcriptional factor were overlapped (p values) and the extent of concordance of the known effects (activation or inhibition) of the targets in the gene lists (Z-score) [45].

## SUPPLEMENTARY MATERIALS AND FIGURES



## References

[1] Ferlay J, Shin HR, Bray F, Forman D, Mathers C, Parkin DM (2010). Estimates of worldwide burden of cancer in 2008: GLOBOCAN 2008. Int J Cancer.

[2] Parkin DM (2001). Global cancer statistics in the year 2000. Lancet Oncol.

[3] Howlader N, Noone AM, Krapcho M, Garshell J, Miller D, Altekruse SF, Kosary CL, Yu M, Ruhl J, Tatalovich Z, Mariotto A, Lewis DR, Chen HS SEER Cancer Statistics Review, 1975-2012. National Cancer Institute.

[4] Seyedin S, Wang PC, Zhang Q, Lee P (2014). Benefit of adjuvant chemoradiotherapy for gastric adenocarcinoma: a SEER population analysis. Gastrointest Cancer Res.

[5] Siegel R, Naishadham D, Jemal A (2013). Cancer statistics, 2013. CA Cancer J Clin.

[6] Deng N, Goh LK, Wang H, Das K, Tao J, Tan IB, Zhang S, Lee M, Wu J, Lim KH, Lei Z, Goh G, Lim QY (2012). A comprehensive survey of genomic alterations in gastric cancer reveals systematic patterns of molecular exclusivity and co-occurrence among distinct therapeutic targets. Gut.

[7] Cancer Genome Atlas Research Network (2014). Comprehensive molecular characterization of gastric adenocarcinoma. Nature.

[8] Shi J, Yao D, Liu W, Wang N, Lv H, He N, Shi B, Hou P, Ji M (2012). Frequent gene amplification predicts poor prognosis in gastric cancer. Int J Mol Sci.

[9] Lee HE, Kim MA, Lee HS, Jung EJ, Yang HK, Lee BL, Bang YJ, Kim WH (2012). MET in gastric carcinomas: comparison between protein expression and gene copy number and impact on clinical outcome. Br J Cancer.

[10] Lennerz JK, Kwak EL, Ackerman A, Michael M, Fox SB, Bergethon K, Lauwers GY, Christensen JG, Wilner KD, Haber DA, Salgia R, Bang YJ, Clark JW (2011). MET amplification identifies a small and aggressive subgroup of esophagogastric adenocarcinoma with evidence of responsiveness to crizotinib. J Clin Oncol.

[11] Graziano F, Galluccio N, Lorenzini P, Ruzzo A, Canestrari E, D'Emidio S, Catalano V, Sisti V, Ligorio C, Andreoni F, Rulli E, Di Oto E, Fiorentini G (2011). Genetic activation of the MET pathway and prognosis of patients with high-risk, radically resected gastric cancer. J Clin Oncol.

[12] Teng L, Lu J (2013). cMET as a potential therapeutic target in gastric cancer (Review). Int J Mol Med.

[13] Smolen GA, Sordella R, Muir B, Mohapatra G, Barmettler A, Archibald H, Kim WJ, Okimoto RA, Bell DW, Sgroi DC, Christensen JG, Settleman J, Haber DA (2006). Amplification of MET may identify a subset of cancers with extreme sensitivity to the selective tyrosine kinase inhibitor PHA-665752. Proc Natl Acad Sci U S A.

[14] Okamoto W, Okamoto I, Arao T, Kuwata K, Hatashita E, Yamaguchi H, Sakai K, Yanagihara K, Nishio K, Nakagawa K (2012). Antitumor action of the MET tyrosine kinase inhibitor crizotinib (PF-02341066) in gastric cancer positive for MET amplification. Mol Cancer Ther.

[15] An X, Wang F, Shao Q, Wang FH, Wang ZQ, Chen C, Li C, Luo HY, Zhang DS, Xu RH, Li YH (2014). MET amplification is not rare and predicts unfavorable clinical outcomes in patients with recurrent/metastatic gastric cancer after chemotherapy. Cancer.

[16] Lai AZ, Cory S, Zhao H, Gigoux M, Monast A, Guiot MC, Huang S, Tofigh A, Thompson C, Naujokas M, Marcus VA, Bertos N, Sehat B (2014). Dynamic reprogramming of signaling upon met inhibition reveals a mechanism of drug resistance in gastric cancer. Sci signal.

[17] Humbert M, Medova M, Aebersold DM, Blaukat A, Bladt F, Fey MF, Zimmer Y, Tschan MP (2013). Protective autophagy is involved in resistance towards MET inhibitors in human gastric adenocarcinoma cells. Biochem Biophys Res Commun.

[18] Gozuacik D, Kimchi A (2004). Autophagy as a cell death and tumor suppressor mechanism. Oncogene.

[19] Yonekawa T, Thorburn A (2013). Autophagy and cell death. Essays Biochem.

[20] Barretina J, Caponigro G, Stransky N, Venkatesan K, Margolin AA, Kim S, Wilson CJ, Lehar J, Kryukov GV, Sonkin D, Reddy A, Liu M, Murray L (2012). The Cancer Cell Line Encyclopedia enables predictive modelling of anticancer drug sensitivity. Nature.

[21] Camidge DR, Bang YJ, Kwak EL, Iafrate AJ, Varella-Garcia M, Fox SB, Riely GJ, Solomon B, Ou SH, Kim DW, Salgia R, Fidias P, Engelman JA (2012). Activity and safety of crizotinib in patients with ALK-positive non-small-cell lung cancer: updated results from a phase 1 study. Lancet Oncol.

[22] Riccardi C, Nicoletti I (2006). Analysis of apoptosis by propidium iodide staining and flow cytometry. Nat Protoc.

[23] Kim YC, Guan KL (2015). mTOR: a pharmacologic target for autophagy regulation. J Clin Invest.

[24] Martina JA, Chen Y, Gucek M, Puertollano R (2012). MTORC1 functions as a transcriptional regulator of autophagy by preventing nuclear transport of TFEB. Autophagy.

[25] Paglin S, Hollister T, Delohery T, Hackett N, McMahill M, Sphicas E, Domingo D, Yahalom J (2001). A novel response of cancer cells to radiation involves autophagy and formation of acidic vesicles. Cancer Res.

[26] Traganos F, Darzynkiewicz Z (1994). Lysosomal proton pump activity: supravital cell staining with acridine orange differentiates leukocyte subpopulations. Methods Cell Biol.

[27] Honscheid P, Datta K, Muders MH (2014). Autophagy: detection, regulation and its role in cancer and therapy response. Int J Radiat Biol.

[28] White E (2012). Deconvoluting the context-dependent role for autophagy in cancer. Nat Rev Cancer.

[29] Amaravadi RK, Lippincott-Schwartz J, Yin XM, Weiss WA, Takebe N, Timmer W, DiPaola RS, Lotze MT, White E (2011). Principles and current strategies for targeting autophagy for cancer treatment. Clin Cancer Res.

[30] Long L, Yang X, Southwood M, Lu J, Marciniak SJ, Dunmore BJ, Morrell NW (2013). Chloroquine prevents progression of experimental pulmonary hypertension via inhibition of autophagy and lysosomal bone morphogenetic protein type II receptor degradation. Circ Res.

[31] Chandra J, Niemer I, Gilbreath J, Kliche KO, Andreeff M, Freireich EJ, Keating M, McConkey DJ (1998). Proteasome inhibitors induce apoptosis in glucocorticoid-resistant chronic lymphocytic leukemic lymphocytes. Blood.

[32] Hong DS, LoRusso P, Hamid O, Beaupre DM, Janku F, Khan R, Kittaneh M, Loberg RD, Amore B, Caudillo I, Hwang YC, Rui Tang R, Ngarmchamnanrith G, Kwak EL (2014). First-in-human study of AMG 337, a highly selective oral inhibitor of MET, in adult patients (pts) with advanced solid tumors. J Clin Oncol.

[33] Aprile G, Leone F, Giampieri R, Casagrande M, Marino D, Faloppi L, Cascinu S, Fasola G, Scartozzi M (2015). Tracking the 2015 Gastrointestinal Cancers Symposium: bridging cancer biology to clinical gastrointestinal oncology. Onco Targets Ther.

[34] Janjigian YY, Tang LH, Coit DG, Kelsen DP, Francone TD, Weiser MR, Jhanwar SC, Shah MA (2011). MET expression and amplification in patients with localized gastric cancer. Cancer Epidemiol Biomarkers Prev.

[35] Yap TA, Olmos D, Brunetto AT, Tunariu N, Barriuso J, Riisnaes R, Pope L, Clark J, Futreal A, Germuska M, Collins D, deSouza NM, Leach MO (2011). Phase I trial of a selective c-MET inhibitor ARQ 197 incorporating proof of mechanism pharmacodynamic studies. J Clin Oncol.

[36] White E (2015). The role for autophagy in cancer. J Clin Invest.

[37] Boya P, Gonzalez-Polo RA, Casares N, Perfettini JL, Dessen P, Larochette N, Metivier D, Meley D, Souquere S, Yoshimori T, Pierron G, Codogno P, Kroemer G (2005). Inhibition of macroautophagy triggers apoptosis. Mol Cell Biol.

[38] Inguscio V, Panzarini E, Dini L (2012). Autophagy contributes to the death/survival balance in cancer photodynamic therapy. Cells.

[39] Jia L, Dourmashkin RR, Allen PD, Gray AB, Newland AC, Kelsey SM (1997). Inhibition of autophagy abrogates tumour necrosis factor alpha induced apoptosis in human T-lymphoblastic leukaemic cells. Br J Haematol.

[40] Nishida K, Yamaguchi O, Otsu K (2008). Crosstalk between autophagy and apoptosis in heart disease. Circ Res.

[41] Yee KS, Wilkinson S, James J, Ryan KM, Vousden KH (2009). PUMA- and Bax-induced autophagy contributes to apoptosis. Cell Death Differ.

[42] Dickstein RJ, Nitti G, Dinney CP, Davies BR, Kamat AM, McConkey DJ (2012). Autophagy limits the cytotoxic effects of the AKT inhibitor AZ7328 in human bladder cancer cells. Cancer Biol Ther.

[43] Cheng T, Roth B, Choi W, Black PC, Dinney C, McConkey DJ (2013). Fibroblast growth factor receptors-1 and -3 play distinct roles in the regulation of bladder cancer growth and metastasis: implications for therapeutic targeting. PLoS One.

[44] Kawakami H, Okamoto I, Arao T, Okamoto W, Matsumoto K, Taniguchi H, Kuwata K, Yamaguchi H, Nishio K, Nakagawa K, Yamada Y MET amplification as a potential therapeutic target in gastric cancer. Oncotarget.2013;.

[45] Choi W, Porten S, Kim S, Willis D, Plimack ER, Hoffman-Censits J, Roth B, Cheng T, Tran M, Lee IL, Melquist J, Bondaruk J, Majewski T (2014). Identification of distinct basal and luminal subtypes of muscle-invasive bladder cancer with different sensitivities to frontline chemotherapy. Cancer Cell.

