# Supplementary material for: Autophagy is required for crizotinib-induced apoptosis in MET-amplified gastric cancer cells
Source: Oncotarget. 2017 Jun 7;8(31):51675–87. doi: 10.18632/oncotarget.18386 (PMC5584279; doi:10.18632/oncotarget.18386)
Supplement: Supplementary file 1 [file oncotarget-08-51675-s001.pdf]

Autophagy is required for crizotinib-induced apoptosis in MET-amplified gastric cancer cells

SUPPLEMENTARY MATERIALS

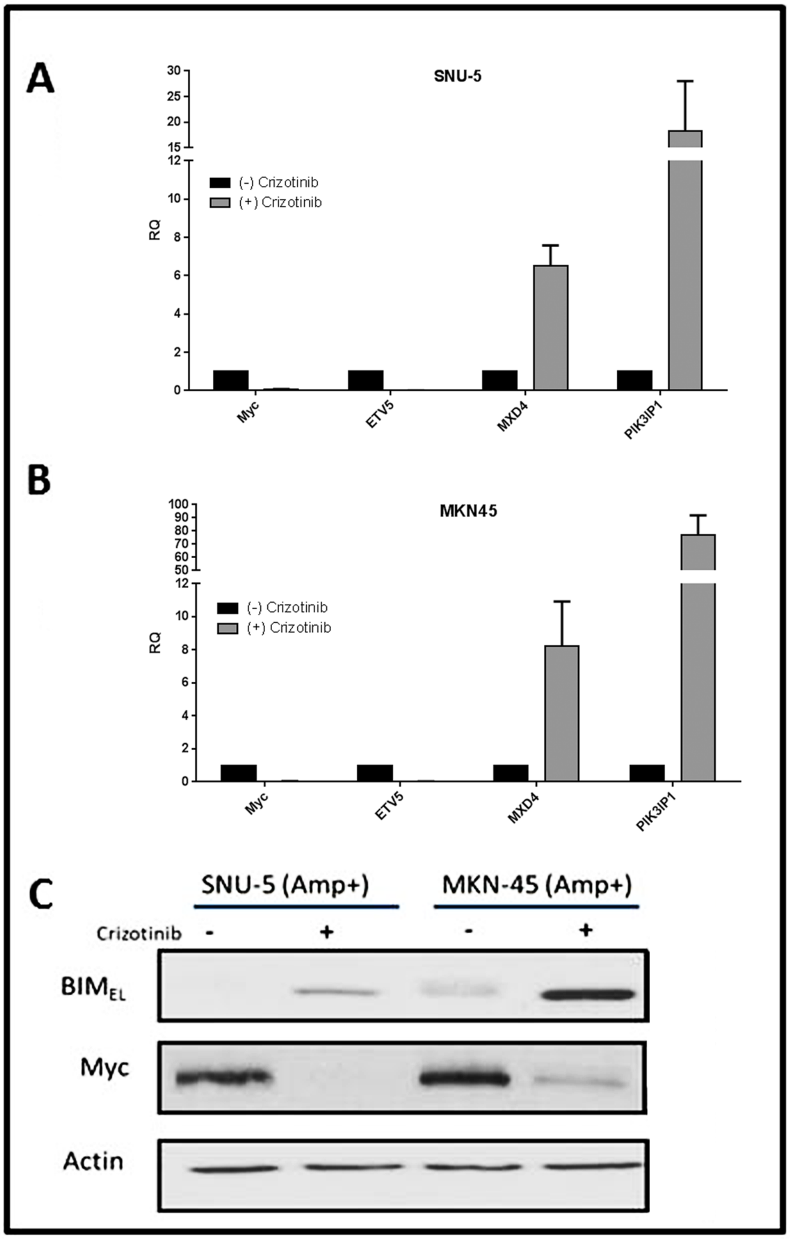

Supplementary Figure 1: Related to Figure 3. Quantitative RT-PCR was used to evaluate the accuracy of the gene expression profiling results. **(A)** Selection of the top downregulated and upregulated genes following treatment with crizotinib in the SNU-5 MET amplified cell line. **(B)** Selection of the top downregulated and upregulated genes following treatment with crizotinib in the MKN45 MET amplified cell line. **(C)** Immunoblotting analysis was used to confirm significant changes in selected up-regulated and downregulated genes at the protein level.

A

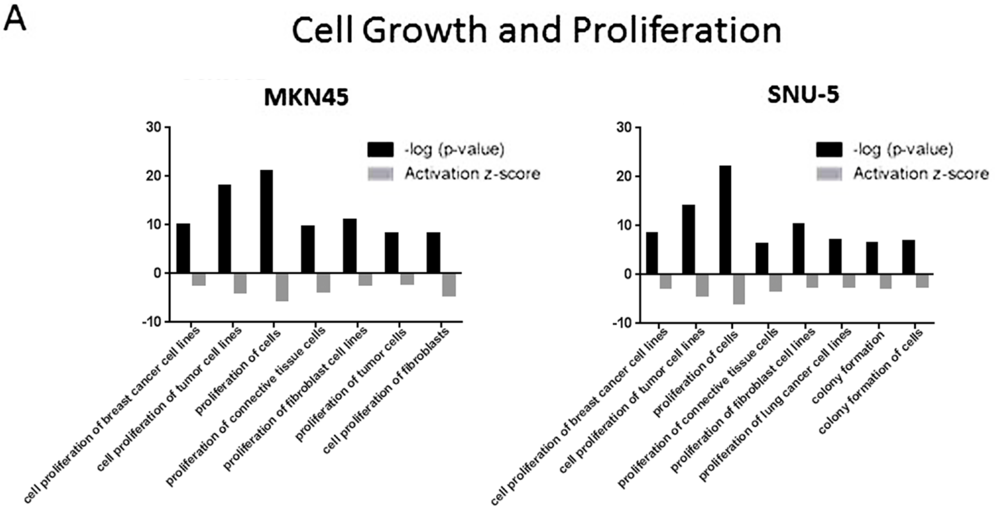

B

SNU-5		
Molecular and Cellular Functions	P-Value	# Molecules
Cellular Growth and Proliferation	4.48E-06 - 9.67E-23	516
Cell Cycle	9.39E-06 - 2.15E-16	263
Cellular Development	2.41E-07 - 1.16E-14	281
Cell Death and Survival	8.96E-06 - 1.02E-12	470
DNA Replication, Recombination and Repair	4.55E-06 - 1.84E-10	151

MKN45		
Molecular and Cellular Functions	P-Value	# Molecules
Cell Cycle	1.77E-06 - 1.34E-24	320
Cellular Growth and Proliferation	1.24E-06 - 8.08E-22	550
Cellular Development	1.24E-06 - 9.69E-19	341
Cell Death and Survival	1.72E-06 - 1.65E-18	524
Cellular Assembly and Organization	1.31E-06 - 4.94E-16	85

Supplementary Figure 2: Related to Figure 4. Additional IPA analysis results. **(A)** Effects of crizotinib on the IPA "cell growth and proliferation" canonical pathway. **(B)** Results of IPA analyses of the top molecular and cellular functions.

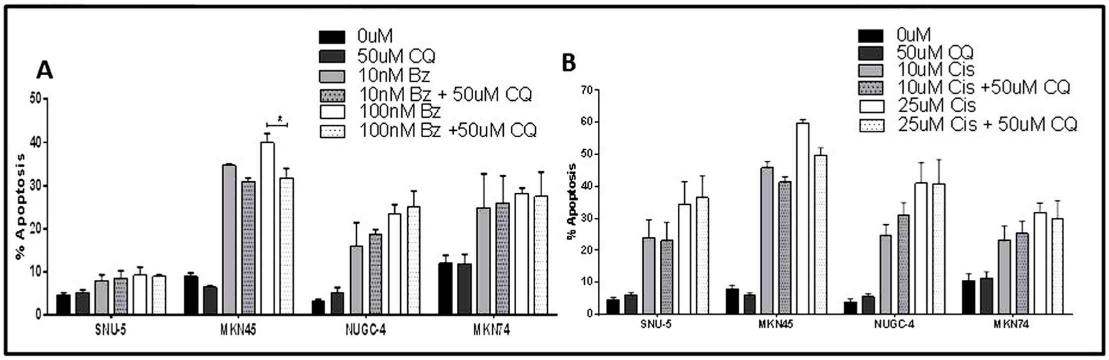

Supplementary Figure 3: Related to Figure 7. Autophagy is not required for apoptosis induced by other agents. **(A)** Effects of chloroquine on bortezomib-induced apoptosis. Gastric cancer cell lines (SNU-, MKN45, MKN74, and NUGC-4) were incubated with 0, 10 or 100nM bortezomib with or without 50uM chloroquine (CQ) for 48hrs hours, and PI/FACS was used to quantify apoptosis. **(B)** Effects of chloroquine on cisplatin-induced apoptosis. Gastric cancer cell lines (SNU-, MKN45, MKN74, and NUGC-4) were incubated with 0, 10 or 25uM cisplatin with or without 50uM chloroquine (CQ) for 48hrs hours, and PI/FACS was used to quantify apoptosis. Data are means ± SEM from three independent experiments. Student t test, *p≤0.05.
